# Spider‐Web and Ant‐Tentacle Doubly Bio‐Inspired Multifunctional Self‐Powered Electronic Skin with Hierarchical Nanostructure

**DOI:** 10.1002/advs.202004377

**Published:** 2021-06-02

**Authors:** Ouyang Yue, Xuechuan Wang, Xinhua Liu, Mengdi Hou, Manhui Zheng, Youyou Wang, Boqiang Cui

**Affiliations:** ^1^ College of Chemistry and Chemical Engineering Shaanxi University of Science & Technology Xi'an Shaanxi 710021 China; ^2^ National Demonstration Center for Experimental Light Chemistry Engineering Education Shaanxi University of Science & Technology Xi'an Shaanxi 710021 China; ^3^ College of Bioresources Chemical and Materials Engineering Shaanxi University of Science & Technology Xi'an Shaanxi 710021 China

**Keywords:** biocompatible and breathable, collagen aggregate nanofibers, multifunctional electronic skin, self‐powered for pressure–humidity–temperature detection, spider‐web and ant‐tentacle doubly bio‐inspired

## Abstract

For the practical applications of wearable electronic skin (e‐skin), the multifunctional, self‐powered, biodegradable, biocompatible, and breathable materials are needed to be assessed and tailored simultaneously. Integration of these features in flexible e‐skin is highly desirable; however, it is challenging to construct an e‐skin to meet the requirements of practical applications. Herein, a bio‐inspired multifunctional e‐skin with a multilayer nanostructure based on spider web and ant tentacle is constructed, which can collect biological energy through a triboelectric nanogenerator for the simultaneous detection of pressure, humidity, and temperature. Owing to the poly(vinyl alcohol)/poly(vinylidene fluoride) nanofibers spider web structure, internal bead‐chain structure, and the collagen aggregate nanofibers based positive friction material, e‐skin exhibits the highest pressure sensitivity (0.48 V kPa^−1^) and high detection range (0–135 kPa). Synchronously, the nanofibers imitating the antennae of ants provide e‐skin with short response and recovery time (16 and 25 s, respectively) to a wide humidity range (25–85% RH). The e‐skin is demonstrated to exhibit temperature coefficient of resistance (TCR = 0.0075 °C^−1^) in a range of the surrounding temperature (27–55 °C). Moreover, the natural collagen aggregate and the all‐nanofibers structure ensure the biodegradability, biocompatibility, and breathability of the e‐skin, showing great promise for practicability.

## Graphical abstract

In this study, a bio‐inspired multifunctional electronic skin (e‐skin) with a multilayer nanostructure based on spider web and ant tentacle is constructed, which can collect biological energy through a triboelectric nanogenerator for the simultaneous detection of pressure, humidity, and temperature. Moreover, the natural collagen aggregate and the all‐nanofiber structure ensure the biodegradability, biocompatibility, and breathability of the e‐skin.

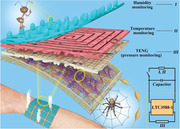

## 1 Introduction

As the largest organ of the human body, skin not only protects the human body from environmental hazards, but also ensures its timely perception of temperature, pressure, and vibration of the external environment.^[^ ^1^, ^2^ ^]^ In the era of Internet of Things, electronic skin (e‐skin) can even surpass the sensory functions of human skin, making it a basic data collection device, with wide range of applications in artificial prostheses, smart robots, wearable devices, health monitoring systems, and other fields.^[^ ^3^, ^4^, ^5^, ^6^ ^]^ Nonetheless, the development of a multifunctional, intelligent, and integrated e‐skin remains a key challenge.^[^ ^7^, ^8^, ^9^, ^10^ ^]^ However, mimicking biological structures (such as human skin, bird feathers, and plant leaves) may aid in the development of materials with excellent performance.^[^ ^11^, ^12^ ^]^ For the practical applications of an e‐skin material, the sensitivity, self‐power capability, biocompatibility, breathability, flexibility, lightness, and cost effectiveness are needed to be assessed and tailored simultaneously.^[^ ^13^, ^14^, ^15^, ^16^, ^17^ ^]^ However, only a few e‐skins endowed with these integrated features have been reported till date.^[^ ^18^, ^19^ ^]^

E‐skins are complex arrays of soft sensors that achieve information by monitoring the conversion of various environmental stimuli, including temperature, humidity, and pressure, into real‐time and visualized electronic pulses.^[^ ^20^, ^21^, ^22^ ^]^ Recently, in order to improve the overall performance of e‐skins, they have been endowed with special functions, such as electroluminescence, self‐healing, shape memory effect, fireproofing, waterproofing, and heat transfer.^[^ ^14^, ^23^, ^24^, ^25^, ^26^ ^]^ Despite the continuous improvement and optimization of the abovementioned multiple functions, e‐skins that can truly imitate human skin and its multiple functions to achieve optimal integration are extremely rare; although this forms the basis for e‐skin to be truly intelligent with widespread applications.^[^ ^7^, ^27^, ^28^ ^]^ Most e‐skins can detect only one type of external stimulus, significantly restricting their practical applications. Some e‐skins are able to detect more than one stimulus but have insufficient sensitivity, as manifested in their small detection range, slow response, and long recovery time.^[^ ^7^, ^27^, ^28^, ^29^ ^]^ For example, in a certain position of an artificial prosthesis such as fingertip, in order to achieve the multistimulation response of human skin, several sensors with different functions must work together. However, the planar array often causes a slight deviation of the sensing position, which is obviously fatal in high‐precision applications, and incorporation of more number of sensors leads to higher manufacturing cost. To address this issue, new conductive or luminescent materials have been developed to increase the sensitivity of e‐skin to pressure, temperature, or humidity, with the need for a complicated synthesis process.^[^ ^24^, ^30^ ^]^ In previous studies, an increased sensitivity was achieved by designing and modifying the structure of the material, such as imitating pyramid or skin‐inspired materials, to obtain a larger contact area under same pressure.^[^ ^31^, ^32^, ^33^ ^]^ However, this method often requires etching or mold inversion, leading to a complicated production process and expensive manufacturing. Therefore, there is an urgent need to develop a multifunctional, sensitive, and truly intelligent e‐skin that can effectively detect pressure, humidity, and temperature, and can provide a basis for the true industrialization of flexible sensing.

Moreover, practically, such a multifunctional e‐skin cannot be completely driven using a traditional battery because the pollution caused by battery electrolyte often causes health hazards and inconvenience associated with its replacement, charging, and recycling.^[^ ^3^, ^34^, ^35^, ^36^ ^]^ Therefore, it is critical to develop a self‐powered sensor array that can harvest human biomechanical energy based on the electrostatic coupling effect.^[^ ^37^, ^38^ ^]^ Although several self‐powered e‐skins have been reported that are tightly attached to the skin to efficiently collect biomechanical energy, most of them are produced with airtight or slightly toxic polymeric membranes such as fluororubber, polydimethylsiloxane, or other compact semiconductor membranes (such as gallium arsenide, titanium dioxide, and zinc oxide).^[^ ^39^, ^40^, ^41^, ^42^ ^]^ These materials may cause skin discomfort and even induce itching and inflammation, in particular, after long‐term contact with human skin.^[^ ^15^, ^43^, ^44^ ^]^ Therefore, it is highly desirable to construct e‐skins using materials with high air permeability and biocompatibility as substrates. Notably, nanofiber (NF) materials with an inherently high surface area and high porosity can provide e‐skin with high sensitivity and excellent air permeability as priority objects.^[^ ^15^, ^45^ ^]^ Besides, considering that the e‐skin should operate normally within a period and then degrade into biocompatible and harmless bio‐electronic products, selection of a substrate material with good biocompatibility is urgently required.^[^ ^46^, ^47^, ^48^ ^]^ Biomaterials such as collagen are used as friction material in triboelectrification because of their ability to lose electrons.^[^ ^49^, ^50^ ^]^ Furthermore, in order to apply biomaterials to e‐skin to significantly harvest biomechanical energy generated by the human body, a reasonable and sophisticated mechanism design is required.

Herein, a multifunctional, self‐powered, sensitive, flexible, breathable, biodegradable, intelligent, and integrated e‐skin with multihierarchical and all‐NFs structure was successfully developed. Further, the e‐skin was used to effectively collect biomechanical energy and monitor whole‐body physiological signals of pressure, temperature, and humidity. The as‐fabricated intelligent integrated e‐skin consists of four parts: 1) a triboelectric (pressure‐sensing) layer composed of collagen aggregate nanofibers (CA NFs) and bead‐chain‐net poly(vinyl alcohol)/poly(vinylidene fluoride) nanofibers (B‐C‐N PVA/PVDF NFs). This layer mimics a spider web and bead‐chain structure and can simultaneously generate electricity by collecting biomechanical energy and detect pressure; 2) Multiwalled carbon nanotubes (MWNTs) doped in poly(3,4‐ethylenedioxythiophene): poly (styrenesulfonate) (PEDOT:PSS) as a conductive material is added to CA, and then spun to obtain CA‐PEDOT‐MWNTs NFs (CA‐P‐M NFs) as a temperature‐sensing layer; and 3) CA and acidified MWNTs are compounded and electro spun to prepare NFs with ant antenna structure (CA‐M NFs) for humidity detection. 4) The triboelectric collection system (LTC3588‐1) was used to collect biomechanical energy and provide energy for temperature and humidity sensing. The pressure sensitivity of the prepared e‐skin reached 0.48 V kPa^−1^ in the range 0–0.5 kPa, 0.25 V kPa^−1^ (0.5–30 kPa), and 0.17 V kPa^−1^ (30–135 kPa). Moreover, it exhibited short response time (2.1 s), recovery time (3.9 s), and excellent temperature coefficient of resistance (TCR = 0.0075 °C^−1^, *R* ^2^ = 0.99) and sensitivity to temperature in the range of 27–55 °C. Even under 25–85% relative humidity (RH), the output signals showed a fabulous linear relationship. Owing to the complete NF structure, e‐skin exhibited an air permeation of 20.87 mm s^−1^, which guarantees comfort during wearing. This study combined multifunctional, sensitive, and self‐powered material to ensure an intelligent and integrated e‐skin in order to detect various physiological or environmental signals. It could even ensure permeability and biodegradability, thereby helping to promote more practical and environmentally friendly applications of e‐skin in human–machine interfaces and artificial intelligence.

## 2 Results

**Figure 1:**
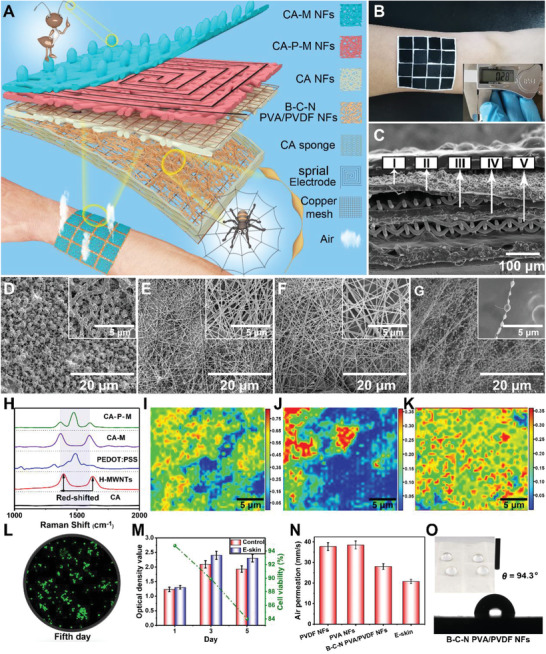
Structure, composition, biocompatibility, and air permeability of e‐skin device: A) Schematic illustration of a 4 × 4 pixels e‐skin. Insets: Partially enlarged view of a single e‐skin pixel. B) Optical photograph of a 4 × 4 pixels e‐skin conformably attached on the arm. The inset is an optical image of e‐skin's thickness. C) SEM image of the cross section of e‐skin (I, II, III, and IV corresponding to D, E, F, and G, and V is copper mesh. D–G) SEM images of CA‐M NFs, CA‐P‐M NFs, CA NFs, and B‐C‐N PVA/PVDF NFs. The inset SEM images show the details separately. H) Raman spectrum of the CA, H‐MWNTs, PEDOT:PSS, CA‐M NFs, and CA‐P‐M NFs. I,J) Raman mapping images of PEDOT:PSS and MWNTs in CA‐P‐M NFs. K) Raman mapping image of MWNTs in CA‐M NFs. L) Photomicrograph of the cell density on the 5th day of the MTT assay of the e‐skin. M) Optical density value and cell viability in MTT test of control and e‐skin. N) The air permeability of e‐skin and its different layers. O) Image of water contact angle of B‐C‐N PVA/PVDF NFs. Inside is an optical image of water droplets resting on B‐C‐N PVA/PVDF NFs.

According to the principle of a triboelectric nanogenerator (TENG), this smart integrated e‐skin can be self‐powered to detect pressure, humidity, and temperature. It also retains sensitivity, breathability, and biodegradability to make it more comfortable and convenient for data collection. **Figure** **1**A demonstrates that in order to improve the sensitivity to external pressure stimuli, B‐C‐N PVA/PVDF NFs were designed as a web‐like structure similar to a spider web. Notably, these web‐like structures help spiders quickly sense subtle vibrations to help them hunt for food efficiently.^[^ ^51^ ^]^ The internal NFs are designed to have a uniform bead‐chain structure, which leads to the increase in the contact area of B‐C‐N PVA/PVDF NFs as the negative friction layer with the CA NFs positive friction layer, thereby significantly increasing the power generation efficiency. Moreover, the sponge also acts as a spring to separate the positive and negative friction layers. More importantly, the electricity collected by the triboelectric (pressure‐sensing) layer through the copper mesh is used for the simultaneous detection of pressure, temperature, and humidity, thereby realizing the versatility, integration, and intelligence of the e‐skin. The CA‐P‐M NFs were designed as the temperature‐sensing layer, whereas the top CA‐M‐NF layer was used as the humidity‐sensing layer. It is well known that as a sensitive biosensor, ant's antennae can perceive humidity, smell, sound, etc.^[^ ^52^ ^]^ Therefore, a humidity‐sensing layer that mimics the antennae of ants was designed herein in order to acquire evenly distributed “antennas” on the surface to achieve high sensitivity to humidity. Among them, the signals of the temperature‐sensing layer and the humidity‐sensing layer can be collected using a spiral electrode.

Figure S1, Supporting Information, shows the manufacturing process of e‐skin, along with a description of the materials and methods used. The all‐NFs network was assembled through a simple layer‐by‐layer electrospinning strategy, allowing the e‐skin to have good air permeability to ensure wearing comfort. Figure 1B displays an optical photograph of the e‐skin attached to the arm. The shown e‐skin consists of 4 × 4 integrated multifunctional sensing units, each with an area of 1 × 1 cm^2^ and used as both a high‐efficiency energy harvester and an intelligent integrated sensitive sensor pixel. The inset in Figure 1B shows the e‐skin with a thickness of only 0.28 mm, which contributes to its softness and breathability. Figure 1C clearly shows the multilayer structure of e‐skin, where I, II, III, and IV correspond to Figures 1D–G, respectively. Figure 1D exhibits that the humidity‐sensing layer is uniformly composed of CA‐M NFs, and the novel “tentacles” are present on the surface. Figure S2A, Supporting Information, displays CA‐M NFs with an average diameter of 180–200 nm. The ordered microstructure of the temperature‐sensing layer composed of CA‐P‐M was observed by scanning electron microscopy (SEM); revealing that the NF diameter is distributed in the range of 180–200 nm (Figure S2B, Supporting Information). Similarly, Figure 1F shows the positive triboelectric layer composed of CA NFs with an average diameter of 140–160 nm (Figure S2C, Supporting Information). The macroscopic spider web structure of B‐C‐N PVA/PVDF NFs as the negative friction layer and the bead‐chain structure of the NFs are shown in Figure 1G and Figure S3, Supporting Information. Figure S2D, Supporting Information, displays the average diameter of the fibers to be 40 and 200 nm, which represent the chains and the beads, respectively. The SEM image of the single bead‐chain structure (inset of Figure 1G) further supports the derivations from Figure S2D, Supporting Information.

Figure S4, Supporting Information, shows the square resistance diagram when CA is doped with different amounts of acidified MWNTs (H‐MWNTs), PEDOT:PSS, and PEDOT:PSS‐MWNTs. With the increase in the amount of doping, the square resistance decreases continuously. Considering the performance and cost of e‐skin, their doping amounts in CA are 12%, 12%, and 10%, respectively. The Raman spectrum (Figure 1H) shows that the red‐shift of the D and G peaks of H‐MWNTs in CA‐M and the red‐shift of pure H‐MWNTs may be caused by the formation of the hydrogen bonds between CA and H‐MWNTs. Moreover, the appearance of D, G, and PEDOT peaks in CA‐P‐M is compared to that in a single CA, which proves the existence of P‐M in CA‐P‐M NFs. Raman mappings (Figure 1I–K) prove the uniform dispersion of PEDOT and H‐MWNTs in CA‐P‐M and H‐MWNTs in CA‐M, ensuring that the material has a continuous conductive path and provides a basis for the stable performance of e‐skin.

The live/dead cell double staining result of the sample on the 5th day in methyl thiazolyl tetrazolium cell proliferation‐toxicity test (MTT assay) showed (Figure 1L) a mass of live cells (green) and fewer dead cells (red). Moreover, the visible absorbance in Figure 1M increased significantly from day 1 to day 3, and decreased on day 5 due to the multiplication of cells from day 1 to day 3 until they were relatively saturated, and from day 3 to day 5, some of the cells began to wither (combined with Figure S5, Supporting Information). On the fifth day, the cell visibility was still as high as 84.3%, which meets the standard of biocompatible materials.^[^ ^53^ ^]^ Figure S6, Supporting Information, shows the natural degradation of e‐skin in the range of temperature and humidity (17 ± 2 °C, 55 ± 5%) without affecting the growth of plant seeds to confirm its degradability.

The air permeability of B‐C‐N PVA/PVDF NFs (28.7%) was lower than those of PVDF NFs (37.6%) and PVA NFs (38.2%) (Figure 1N) because the bead‐chain structure slightly reduced the porosity of the material. The air permeability of e‐skin was decreased to a certain extent due to the superposition of multilayer materials, which improved the complexity of the gas transmission path. However, it was still as high as 20.7% that could achieve the purpose of wearing comfort.^[^ ^54^, ^55^ ^]^ Figure 1O shows that the B‐C‐N PVA/PVDF NFs, as the bottom layer, exhibit moderate hydrophobicity (water contact angle of 94.3°), which ensures not only the wearing comfort of the e‐skin, but also the damage‐revention of the structure of the e‐skin due to excessive sweat, thereby reducing its service life.

**Figure 2:**
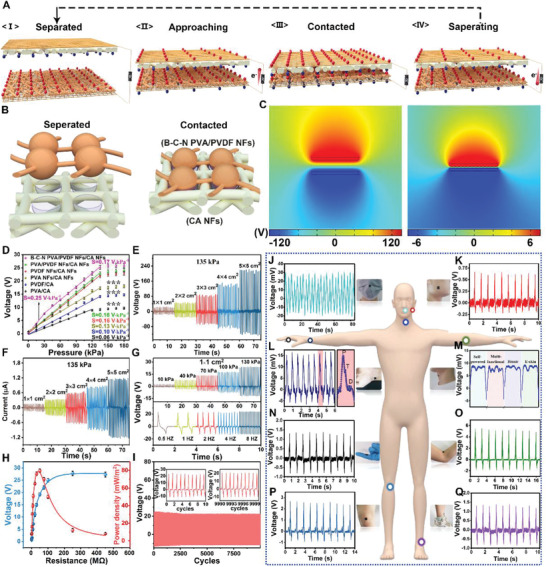
The performance of triboelectric (pressure‐sensing) layer: A) Schematic illustration of the working principle of triboelectric (pressure‐sensing) layer as a TENG. B) Schematic representation of CA NFs and B‐C‐N PVA/PVDF NFs contact separation process. C) Finite element simulation of potential distribution at different separation distances (5 and 0.1 mm) between CA NFs and B‐C‐N PVA/PVDF NFs. D) The output voltage and sensitivity of e‐skin pixels (1 × 1 cm^2^) with different materials and structure. The variation is represented by the standard deviation of three independent replicates in all graphs, *** (*p*‐value < 0.05). E,F) Output voltage and current of the triboelectric (pressure‐sensing) layer, with different sizes as a pixel. G) The output voltage of e‐skin pixel (1 × 1 cm^2^) at different pressures and frequencies. H) The relationship between the output voltage and power density of triboelectric (pressure‐sensing) layer and the external load resistance. I) The output stability of the e‐skin pixel (1 × 1 cm^2^) was tested at 130 kPa for 10 000 cycles. The internal graph is the signal graphs of the first ten cycles and the last ten cycles. J–Q) Signals of whole‐body monitored using the e‐skin attached to different parts, which are marked by circles in different colors in the human body model.

As a typical contact separation type TENG, when the friction layers of e‐skin underwent a continuous contact separation process (**Figure** **2**A), alternating potential and current were generated due to contact electrification and electrostatic coupling, achieving power generation and pressure detection. Figure 2B reveals that the contact between the spherical shape of B‐C‐N PVA/PVDF NFs and the holes formed by the CA NFs produces a large contact area, which is conducive to the improvement of the power generation efficiency. The electrostatic finite element simulation was carried out to narrate the mechanism of the TENG. Furthermore, the numerical calculation of the potential distribution result of contact/separation process of the triboelectric layer was depicted (Figure 2C), which concluded the increase in the potential difference from 12 to 240 V. Figure 2D and Figure S7, Supporting Information, show the performance of a 1 × 1 cm^2^ pixel composed of B‐C‐N PVA/PVDF NFs and CA NFs, indicating a higher sensitivity (S = 0.48 and 0.25 V kPa^−1^ in the range of 0–0.5 and 0.5–30 kPa, respectively) than other structures presented in this Figure (*S* ≤ 0.16 V kPa^−1^) under pressure due to the presence of the spider web structure, which resulted in significant improvement of the sensitivity. Moreover, the sensitivity was still excellent (0.17 V kPa^−1^ in the range of 30–135 kPa) compared to that reported in some distinguished studies (Table S1, Supporting Information), which is attributed to the higher contact area provided by the bead‐chain structure. In addition to the structural design, the size of a single pixel also exhibited a great impact on the output performance of the e‐skin. When the pixel area was 5 × 5 cm^2^, the voltage and current could reach 220 V and 1.12 µA, respectively (Figure 2E,F). Considering that the pixel size is too large to adversely affect the flexibility of the e‐skin, the pixel area used in this study was 1 × 1 cm^2^, and its voltage and current could reach 26 V and 0.19 µA, respectively (Figure 2F,G). Moreover, its output voltage was found to be proportional to the applied pressure (Figure 2G). Furthermore, a single e‐skin pixel has excellent stability to different frequency stimuli under a pressure of 100 kPa (Figure 2G). Figure S8, Supporting Information, shows that the maximum test frequency of e‐skin signal is 19 Hz in order to prevent the signals from distortion. Video S1, Supporting Information, shows good dynamic responsiveness of a pixel at lower frequencies.

By loading different external resistors (0–450 MΩ), the power output performance of the e‐skin was systematically investigated (Figure 2G). The output voltage proliferated with the change in the resistance from 0 to 150 MΩ, and then saturated (26.5 V) with the further increase in the resistance. With an external load resistance of 47 MΩ, the maximum load peak power reached 79.4 mW m^−2^. The delivered power was able to fully drive some small electronic devices, so as to provide this intelligent integrated e‐skin with the energy to detect temperature and humidity in an efficient and reliable manner.

Durability is an essential influencing factor for practical applications of e‐skin. Therefore, the e‐skin was tested under the external circulation pressure of 0/100 kPa. After 10 000 cycles, the voltage output attenuated to only 3.5%, demonstrating the durability of the intelligent integrated e‐skin (Figure 2I). Besides, in order to fully prove the sensitive response of e‐skin to the subtle movements of the human body and the comprehensive detection of human muscle activity, clinical trials are necessary. The e‐skin can be used to display sensitive signals corresponding to human breathing (Figure 2J), language (Figure 2M), and wrist pulse (Figure 2L), wherein the characteristic peaks of P, T, and D of the heartbeat are clearly captured. Moreover, Figure 2M also shows that, when the human throat vibrates in order to speak, e‐skin can even sharply capture the phonetic transcription of every word. Similarly, e‐skin plays an important role in detecting body muscle movement. After scientific research, e‐skin produces unique output signals for the movements attached to different parts of the body, such as cheek muscle movement during chewing (Figure 2K); bending of fingers (Figure 2N); and movement of the elbows (Figure 2O), knee joints (Figure 2P), and ankles (Figure 2O) during running. Figure S9, Supporting Information, displays excellent performance of e‐skin under different pressures caused by the tester running at different speeds (5, 10, and 20 km h^−1^ corresponding to ≈90, 110, and 130 kPa, respectively). Consequently, e‐skin can generate specific signals for the movement of the abovementioned different positions, and accurately reflect the intensity of the exercise. Moreover, the anti‐interference ability of pressure‐sensing layer in the complex environment provides the basis for its wide range of applications (Figure S14, Supporting Information).

For a better investigation of the practical applications of the e‐skin, a series of experiments was designed. First, as shown in Figure S10 and Video S2, Supporting Information, the e‐skin output current could be easily used for real‐time spatial mapping through the rectifier bridge in response to the touch of the visible LED signal on the board. Thereafter, a small ball weighing 30 g was arranged from “11” pixels to “44” pixels through “22” and “33” pixels, respectively (Figure S11a, Supporting Information). After data collection, Figure S11B,C, Supporting Information, shows that the corresponding signals on the pixel were attributed to the rolling track of the ball, with no signal delay. Figure S12A, Supporting Information, shows that a single e‐skin pixel can fully charge a commercial capacitor (16 V, 1000 µF) in 4800 s under certain conditions (90 kPa, 5 HZ). The capacitor can endow the e‐skin with property to detect the temperature and humidity and provide energy for more than 60 s of normal operation whenever required (Figure S12B, Supporting Information).

**Figure 3:**
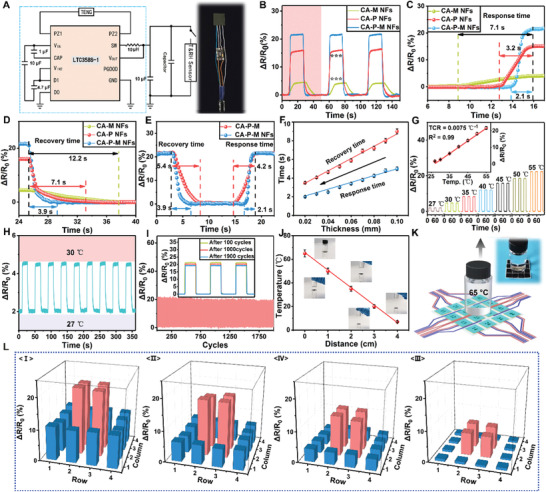
The temperature detection performance of multifunctional intelligent integrated e‐skin at 45% RH: A) Power management circuit (LTC3588‐1). Inset is an optical image of an integrated system of e‐skin pixels (1 × 1 cm^2^) and energy management system. B) Normalized relative resistance variation (Δ*R*/*R*_0_) of temperature‐sensing layer of different components (CA‐M NFs, CA‐P NFs, and CA‐P‐M NFs) from 25 to 55 °C. The variation is represented by the standard deviation of three independent replicates in all graphs, *** (*p*‐value < 0.05). C,D) The response and recovery time corresponding to the first cycle in (B). E,F) Response and recovery time of different structures and thicknesses. G) The output signals of the temperature‐sensing layer at different temperatures. Inset is TCR values for temperature‐sensing layer. H) The instantaneous electrical response of the temperature‐sensing layer when the temperature changes in limited range (27–30 °C). I) Temperature cycling test for the e‐skin for 0−2000 cycles. Inset shows the signals after 100, 1000, and 1900 cycles. J) Distance‐dependent receiving temperature of e‐skin. K) The dynamic experimental simulation diagram of the e‐skin gradually moving away from the heat source (65 °C). The interior is an optical photo of the experiment. L) The matrix data map corresponds to the number of e‐skin pixels for the dynamic experiment in (K) at different distances (1, 2, 3, and 4 cm) collected by using LabVIEW.

In order to achieve an intelligent and integrated e‐skin, the collected biological energy must be recycled to the e‐skin itself, an imperative issue that has been ignored thus far. Herein, a commercial power management circuit (LTC3588‐1) (**Figure** **3**A) was applied to the power management and application of e‐skin, converting the AC voltage output of the triboelectric (pressure‐sensing) layer of e‐skin to the DC voltage output of 3.3 V. The power management circuit incorporated a low‐loss full‐wave rectifier and a high‐efficiency step‐down converter, providing a complete energy collection solution with high output impedance for the e‐skin's triboelectric layer and continuous power supply for temperature and humidity sensing (inset in Figure 3A). In order to obtain an intelligent e‐skin, responsiveness to temperature is an important factor that must be considered, and the response time and temperature response range are key points affecting its performance. Figure 3B exhibits the signals of CA‐M NFs, CA‐P NFs, and CA‐P‐M NFs with a single pixel (1 × 1 cm^2^) at 50 °C for multiple cycles. CA‐P‐M NFs showed a stronger signal (Δ*R*/*R*_0_ = 54.5%) than CA‐M NFs (11.2%) and CA‐P NFs (39.5%) under the same temperature conditions. Figure S13, Supporting Information, illustrates that the structure of all the NFs facilitates the transfer of temperature through the medium, which changes the conductivity of P‐M and thus displays different signals. In order to study the response time and recovery time under the same conditions, three cycles were performed as displayed in Figure 3B and a cycle was selected for detail description (Figure 3C,D). Figure 3C shows that CA‐P‐M NFs exhibit a shorter response time (2.1 s) than CA‐M NFs (7.1 s) and CA‐P NFs (3.2 s). Similarly, Figure 3D also illustrates the excellent recovery speed of CA‐P‐M NFs (3.9 s). Abovementioned results show the reaction time of e‐skin to high temperature (50 °C); and often, the time required at low temperatures is shorter. The roots of the abovementioned advantages are: 1) temperature‐sensitive PEDOT:PSS is used as the conductive channel material. 2) H‐MWNTs enhance carrier hopping and tunneling conduction in temperature sensor layer (compare Figure S4, Supporting Information).^[^ ^43^, ^56^ ^]^ Moreover, compared to CA‐P‐M film, the signal intensity of CA‐P‐M NFs at the same temperature shows little change due to the use of same raw materials (Figure 3E). However, CA‐P‐M NFs show a microporous structure between NFs that is more convenient for heat penetration. This structure not only helps heat in the external environment to enter the temperature‐sensing layer, thereby increasing its response sensitivity, but also offers recovery benefits.

Of course, the thickness of the temperature‐sensing layer also affects the performance. With the decrease in the thickness of the layer, the response time and recovery time become shorter (Figure 3F). Considering the controllability and cost of preparation, thickness of 0.02 mm was selected as the best condition herein. The temperature‐sensing layer shows a larger detection range of 27–55 °C, which can fully meet the needs of e‐skin for temperature detection (Figure 3G). TCR is utilized to evaluate the sensitivity of the thermal response and is defined as TCR = [(*R*_T_ − *R*_0_)/*R*_0_)]/*δT*, where *R*_T_ is the instantaneous resistance under measured temperature and *R*_0_ is original resistance.^[^ ^57^, ^58^ ^]^ The performance (TCR = 0.0075 °C^−1^) is also excellent compared to that of other professional flexible temperature sensors (Table S2, Supporting Information). Furthermore, Figure 3H displays the characteristics of the output signals in the temperature range of 27–30 °C. The signal was stable at 5% and the fluctuation range was inconspicuous, which proves the stability of the temperature‐sensing layer for temperature detection. Furthermore, the excellent durability of the temperature‐sensing layer is also required as part of the e‐skin. The signal of the temperature‐sensing layer remained relatively stable during 2000 cycles of 25/55 °C (Figure 3I). Moreover, comparative analysis of the signals corresponding to 100, 1000, and 1900 cycles (inset Figure 3I) indicated that the signals were observed to have less than 2% attenuation, undoubtedly ensuring the long‐term effective and normal operation of e‐skin. Non‐interference is a critical factor for multifunctional e‐skin. Figure S15, Supporting Information, proves that, under different humidity and pressure conditions, the responsiveness of e‐skin to temperature was constant.

The temperature transmission in the medium decreases gradually with the increase in distance, and the temperature of the heat source acquired by the e‐skin gets affected by the increase in distance. Thus, a sample bottle filled with water at 65 °C was used as the heat source and the relationship with respect to the distance between the heat source and e‐skin was tested (Figure 3J). With the increase in the distance, the temperature acquired by the e‐skin was lower. In order to more clearly prove the specific relationship between the real‐time signal of e‐skin and the heat source, the heat source was designed to be 1, 2, 3, and 4 cm away from the marked e‐skin, and the signal of the e‐skin was detected (Figure 3K). With the increase in the distance, the signal generated by the e‐skin dropped significantly (Figure 3L,J). Moreover, the pixels of the e‐skin generated different signals due to the different distances from the heat source. Through such signals, the e‐skin could accurately simulate the shape of the heat source, endowing it with more efficient intelligence.

**Figure 4:**
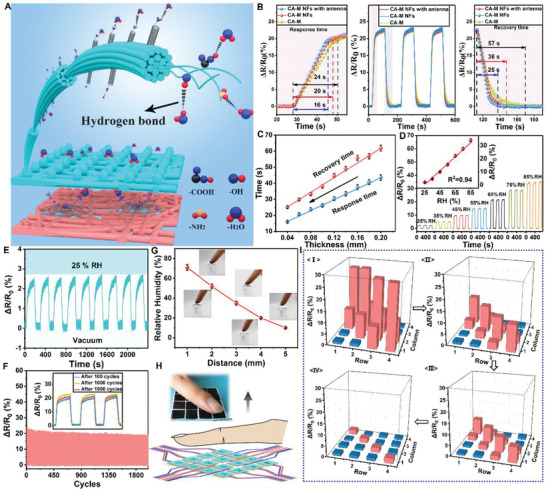
The sensing performance of e‐skin for humidity at 25 °C: A) The mechanism of humidity‐sensing layer to humidity. B,C) Effects of different structures and thickness on humidity sensitivity of humidity‐sensing layer. D) Response signals of e‐skin at different relative humidity. The internal diagram illustrates the linear relationship between the output signal and RH. E) Nine cycles of e‐skin at different humidity (0–25%). F) The fluctuation of the signals of e‐skin during 2000 cycles at a certain RH (65%). The internal picture is the instantaneous signal of e‐skin after 100, 1000, and 1900 cycles. G) The relationship between the humidity received by the e‐skin and the distance from fingertip. H) The illustration image of fingers gradually moving away from the numbered e‐skin. Inside is an optical image of the experiment. I) The matrix data map corresponds to different distances (1, 2, 3, and 4 mm) in (H).

Human skin has a keen sense of pressure and temperature, yet performs poorly in the perception of humidity because the skin lacks humidity‐sensitive receptors. As an intelligent integrated e‐skin, humidity detection is indispensable in its versatility, in order to make up for the defects of natural skin as well as to facilitate the accurate analysis of the surrounding environment. However, the currently used multifunctional sensors lack in humidity detection, manifested by extremely long response and recovery times, a small detection range, and poor material biocompatibility. Considering the abovementioned shortcomings, a novel bionic “antenna” that mimics the antennae of ant was designed on the surface of CA‐M NFs (Figures 1A and **4**A). It can significantly increase the contact area of water molecules with the layer based on the NF structure, which is conducive to the penetration and escape of water molecules. Several ─NH_2_, ─COOH, and ─OH groups provided by CA, H‐MWNTs, and glycerin have a hydrophilic effect of hydrogen bonds, which benefit the humidity‐sensing layer to capture the water molecules in a certain RH environment, significantly improving the response to humidity (Figure 4A). Moreover, the temperature‐sensing layer was designed to have a certain waterproof performance by adding sodium methyl silicate water repellent, ingeniously avoiding excessive moisture from entering the e‐skin and resulting in a shorter recovery time. CA‐M‐NFs with “antenna” showed the shortest response time (16 s) and recovery time (25 s) compared to CA‐M‐NFs (20 and 36 s) and CA‐M (24 and 56 s) (Figure 4B). This result is attributed to the large number of channels provided by the NF structure, accelerating the movement of water molecules in the humidity‐sensing layer. Furthermore, the “antenna” structure on the NFs surface not only makes the channel more uniform and capaciousness due to the spatial effect, but also acts as a “receptor” on the surface of the humidity‐sensing layer to increase the sensitivity to humidity. In addition, the thickness of the humidity‐sensing layer can directly affect the diffusion and residence time of water molecules, thereby directly affecting the sensitivity of e‐skin to humidity. With the decrease in the thickness, the response time and recovery time of the e‐skin to humidity decreased (Figure 4C). Considering that the sample was not uniform when it was too thin during the spinning process, it adversely affected the durability of the e‐skin. The thickness of humidity‐sensing layer prepared in this study was 0.04 mm.

As part of the intelligent integrated e‐skin, the humidity‐sensing layer should also have a wide dynamic detection range and high sensitivity to humidity. To test the performance, a pixel was selected from e‐skin to evaluate the humidity at 25 °C. The detection range of e‐skin for humidity was 25–85% RH (Figure 4D), which is quite high compared to that for other humidity sensors (Table S3, Supporting Information). Moreover, the output signal and humidity have an obvious linear relationship within this range (inset Figure 4D). The repeated response to 25% RH with nine cycles showed great stability and rapid response and recovery. The experiment was reproduced well through a stable signal. Furthermore, temperature and pressure showed an acceptable effect on humidity sensing, which also ensured normal functioning of e‐skin in complex environments (Figure S16, Supporting Information). Figure S17, Supporting Information, illustrates that the compensation of different signals is calculated through the analysis of a large amount of experimental data, which can separate complex signals and thus accurate detection results can be obtained. In order to verify the durability of the humidity‐sensing layer, 2000 cycles were performed under the conditions of 75% RH and 25 °C. During the cycles, the output signal of e‐skin produced only 3.8% signal degradation (Figure 4F); however, its stability supported long‐term effective operation.

To demonstrate non‐contact humidity sensing of e‐skin, a finger was placed on the flexible device and the humidity that the pixel received was measured. With the increase in the distance between the fingertip and the pixel from 1 to 5 mm, the humidity detected by the pixel decreased from 72% to 9.8%. To clearly demonstrate the overall responsiveness of the intelligent integrated e‐skin to the external environment, the finger was placed at different distances from numbered e‐skin device (Figure 4H). When the finger moved away, the humidity detected by the e‐skin sharply decreased and the intensity of displayed signals became weaker (Figure 4I). Moreover, the silhouette and distance of the finger could also be displayed based on signal analysis. These results also demonstrate the ability of e‐skin to recognize the external environment, which is conducive to the applicability and intelligence of skin. Moreover, the sensor shows smaller errors compared to commercial sensors, which pave the way for its practical applications (Figure S18, Supporting Information).

## 3 Conclusion

An intelligent integrated e‐skin was developed, which was self‐powered and able to detect pressure, temperature, and humidity. Moreover, the application of biocompatible CA, and all NFs structure provided good biocompatibility and air permeability (air permeation 20%). In the detection range of 0–0.5 and 0.5–30 kPa, the detection sensitivity was 0.48 and 0.25 V kPa^−1^, respectively, and 0.17 V kPa^−1^ within 30–135 kPa, owing to its spider‐web structure and internal bead‐chain fiber structure. Furthermore, the energy collected by the triboelectric (pressure‐sensing) layer was transferred to the e‐skin to allow for the temperature and humidity detection. In the dynamic range of 25–55 °C, the e‐skin presented a good thermal response (0.0075 °C^−1^, *R* ^2^ = 0.99). Similarly, the water absorption performance of the CA material and the ant antennae mimicking structure indicated that the addition of an “antenna” to the humidity‐sensing layer of the e‐skin could provide a good humidity response at 25–85% RH (response time is 16 s, recovery time is 25 s). Based on the abovementioned multiple advantages, the excellent self‐powered, multifunctional, and low‐cost integrated intelligent e‐skin can be widely used in the fields of intelligent robots, interactive wearable devices, artificial prostheses, and disabled patients.

## 4 Experimental Section

### Fabrication of Poly(vinyl alcohol)/Poly(vinylidene fluoride) Nanofibers

PVA (Mw = 27000, Borgneni) was dissolved in deionized water at 90 °C after stirring for 2 h at a concentration of 7 wt%. The homogeneous dispersion liquid of PVA/PVDF (15 wt%) was prepared by stirring for 20 h following the addition of PVDF powders (8 wt%) (Mw = 40000, Macklin). A copper mesh (0.07 mm thick, Tiejin) was fixed on the collector 15 cm away from the needle to be evenly covered by PVA/PVDF NFs. The electrospinning machine (Nuohai Life Science) was maintained under constant spinning conditions (25 ± 2 °C, 45 ± 5% RH, 25 kV) during the working process, at a feed rate of 1.5 mL h^−1^. Finally, the samples were placed in an oven at 60 °C for 5 h to remove the residual solvent.

### Fabrication of Collagen Aggregate Nanofibers and Assembled Triboelectric (Pressure‐Sensing) Layer

CA (10 wt%, extracted in the laboratory) was dissolved in hexafluoro‐2‐propanol (HFIP, Aladdin) by stirring for 30 min at 40 °C. Electrospinning was carried out at a feed rate of 2 mL h^−1^, applied voltage of 30 kV, a collector (copper mesh)‐spinner distance of 20 cm (the optimization scheme is shown in Figure S19, Supporting Information), and constant environmental conditions (25 ± 2 °C, 45 ± 5% RH). Aqueous solution of CA (30%) was dried in a freeze dryer for 24 h until a sponge was formed, and cut into a 1 × 1 cm ring with a thickness of 0.1 µm (ring width 0.1 cm). CA NFs, PVA/PVDF, and the CA sponge were assembled according to Figure S1, Supporting Information, to obtain a triboelectric (pressure‐sensing) layer.

### Fabrication of Temperature‐Sensing Layer

MWNTs (0.3 wt%) were added to the PEDOT:PSS (1.3 wt%) solution, mixed thoroughly by ultrasonication for 2 h, and collagen aggregate (15 wt%) was added, followed by stirring for 15 h at 40 °C. Finally, the samples were dried at 50 °C for later use. CA‐P‐M (10 wt%) was dissolved in HFIP after stirring for 10 h, 5 wt% of sodium methyl silicate water repellent (DOW Corning) was added, and stirring was continued for 20 h to obtain a uniformly mixed CA‐P‐M spinning solution. The assembled triboelectric (pressure‐sensing) layer was placed on the collector and the prepared CA‐P‐M spinning solution was placed in a plastic syringe, and the needle was placed 20 cm away from the collector. Voltage was maintained at 30 kV and feeding rate at 2 mL h^−1^ (the optimization scheme is shown in Figure S20, Supporting Information). The obtained sample was dried at 70 °C for 2 h to remove the remaining solvent, then placed on a platform, and graphite was sprayed on it with a spray gun to form a circular cross electrode (ring width 0.07 cm), thus affording sample (a).

### Fabrication of Humidity‐Sensing Layer

H‐MWNTs (1.2 wt%) were ultrasonically dispersed in deionized water for 1 h and then CA (10 wt%) was added. The contents were stirred at 40 °C for 15 h and dried in a vacuum oven for 12 h to obtain CA‐M. CA‐M (10 wt%) was dissolved in HFIP under the same conditions as mentioned above, and glycerin (5%) was added. The contents were stirred vigorously for 24 h, and then placed in the syringe of the spinning machine. Sample (a) was attached to the collector, 10 cm away from the needle, the voltage was 18 kV, and constant environmental conditions (50 °C, 10% RH) were maintained (the optimization scheme is shown in Figure S21, Supporting Information). The resultant samples (pixels of e‐skin) were dried in an oven at 60 °C for 5 h to remove residual solvents.

### Electronic Skin Assembly

The positive and negative poles of triboelectric (pressure‐sensing) layer were connected to the input ports of the power management system to collect the energy generated by the movement. The output terminal of the energy management circuit was piped with the cross electrode of the e‐skin, as an energy source for humidity and temperature detection. The prepared e‐skin was tightly attached to human skin using the double‐sided adhesive tapes to sensitively acquire pressure, temperature, and humidity information.

### Characterizations and Measurements

The morphology of NFs and the cross section of composite membranes were characterized by SEM (FEI Verios 460 SEM, FEI, America). The chemical components were analyzed by Raman spectroscopy using a DXRxi Raman imaging microscope with a 532‐nm laser (THEM, America). The square resistance was measured using an RTS‐4 four‐probe square resistance meter (SANNUO, China). The air permeability, which represents the breathability performance, was tested using a densometer (Labthink, China). The water contact angle was examined using a contact angle analyzer (LAUDA, Germany). The electrical signals were obtained using a Keithley 2635B source meter controlled by a LabView‐based data acquisition system. The real‐time signals of humidity and temperature sensing were measured under a constant voltage of 3.3 V. The humidity and temperature were maintained in a HWS‐150B constant temperature and humidity cabinet (TAISETE, China). The statistical product and service solutions (SPSS 19.0) were used for statistical analysis, and the data were expressed as mean ± standard deviation (x ± s). Sample size (*n*) for each statistical analysis was 4. Single factor analysis of variance was used, and a *p* value of <0.05 indicated a significant difference.

## Conflict of Interest

The authors declare no conflict of interest.

## Supporting information

Supporting Information

Supplemental Video 1

Supplemental Video 2

## Data Availability

The data used to support the findings of this study are available from the corresponding author upon request.
