# Supplementary material for: Spider‐Web and Ant‐Tentacle Doubly Bio‐Inspired Multifunctional Self‐Powered Electronic Skin with Hierarchical Nanostructure
Source: Adv Sci (Weinh). 2021 Jun 2;8(15):2004377. doi: 10.1002/advs.202004377 (PMC8336620; doi:10.1002/advs.202004377)
Supplement: Supplementary file 1 — Supporting Information [file ADVS-8-2004377-s002.pdf]

© 2021 Wiley-VCH GmbH

ADVANCED SCIENCE
Open Access

# Supporting Information

for *Adv. Sci.,* DOI: 10.1002/advs.202004377

# Spider-web and Ant-tentacle Doubly Bio-inspired Multifunctional Self-powered Electronic Skin with Hierarchical Nanostructure

*Ouyang Yue[a, c], Xuechuan Wang[a, c,*], Xinhua Liu[b, c,*], Mengdi Hou[a, c], Manhui Zheng[b, c], Youyou Wang[b, c], Boqiang Cui[b, c]*

# Supporting Information

# Spider-web and Ant-tentacle Doubly Bio-inspired Multifunctional Self-powered Electronic Skin with Hierarchical Nanostructure

*Ouyang Yue*[a, c], *Xuechuan Wang*[a, c,*], *Xinhua Liu* [b, c,*], *Mengdi Hou*[a, c], *Manhui Zheng*[b, c] , *Youyou Wang*[b, c], *Boqiang Cui*[b, c]

[a] College of Chemistry and Chemical Engineering, Shaanxi University of Science & Technology, Xi'an 710021, Shaanxi, China

[b] College of Bioresources Chemical and Materials Engineering, Shaanxi University of Science & Technology, Xi'an 710021, Shaanxi, China

[c] National Demonstration Center for Experimental Light Chemistry Engineering Education, Shaanxi University of Science &Technology, Xi'an 710021, Shaanxi, China

*Corresponding author: wangxc@sust.edu.cn; liuxinhua@sust.edu.cn;

Figure S1. Preparation process of intelligent integrated e-skin

Figure S2. (A) Diameter distribution of the CA-M NFs. (B) Diameter distribution of the CA-P-M NFs. (C) Diameter distribution of the CA NFs. (D) Diameter distribution of the Bead-Chain-Net PVA/PVDF NFs.

Figure S3. (A) SEM image of Bead-Chain-Net PVA/PVDF nanofibers.

Figure S4. Sheet resistance of CA-M NFs, CA-P NFs and CA-P-M NFs after doping with different proportions of MWNTs, PEDOT:PSS and PEDOT-MWNTs.

Figure S5. Photomicrograph of the cell density of the control (A, C and E) and e-skin (B, D) in MTT test on the first day (A, B), the third day (C, D), and the fifth day (E).

Figure S6. The natural degradation of e-skin.

Figure S7. The signals of pressure-sensing layer under light pressure. (B)The sensitivity of e-skin pixels (1 × 1 $cm^2$) under light pressure.

Figure S8. The signals of e-skin at 19 HZ.

Figure S9. The signals of e-skin under high pressure: (A) The diagram of e-skin attached to the arch of foot to test the performance under different pressures caused by the tester running at different speeds (5 km $h^{-1}$,10 km $h^{-1}$, and 20 km $h^{-1}$ correspond approximately to 90 kPa, 110 kPa, and 130 kPa, respectively). (B) The signals of e-skin when the tester run at speed of 5 km $h^{-1}$. (C) The signals of e-skin when the tester run at speed of 10 km $h^{-1}$. (D) The signals of e-skin when the tester run at speed of 20 km $h^{-1}$.

Figure S10. (A) Sample photos of commercial bridge rectifiers. (B). Circuit schematic diagram of commercial bridge rectifier. (C). Photo of the current produced by the e-skin driving the LED after being processed by a commercial bridge rectifier.

Figure S11. (A) An explanatory drawing of the movement of a 30 g ball in e-skin from "11" to "44". Optical photo inside. (B and C) Corresponding signals during the

movement of the ball.

Figure S12. A single e-skin pixel charges a commercial capacitor (16 V, 1000 μF) under certain conditions (90 kPa, 5 HZ). (B) The capacitor acted as the power source for the humidity and temperature detection of e-skin.

Figure S13. The mechanism of temperature-sensing layer to temperature.

Figure S14. (A) The influence of the pressure-sensing layer under different relative humidity at 25°C. (B) The influence of the pressure-sensing layer under different temperature at 45% RH.

Figure S15. (A) The influence of the temperature-sensing layer under different pressures at 45% RH. (B) The influence of the temperature-sensing layer under different humidity at 130 kPa.

Figure S16. (A) The influence of the humidity-sensing layer under different temperature at 130 kPa. (B) The influence of the humidity-sensing layer under different pressure at 25°C.

Figure S17. (A) Illustration of e-skin tested in a constant temperature and humidity cabinet. (B) The signals fitting and compensation of temperature sensor layer at different humidity. (C) (E) The signals of e-skin in complex conditions (25/55°C, 45/85% RH, and 0/130 kPa). (D) Temperature and humidity signals after separation and compensation.

Figure S18. (A) The photo images of e-skin being tested in a constant temperature and humidity box under specific conditions (45.4°C, 45% RH). (B) The instantaneous electrical response of the temperature-sensing layer. (C) The corresponding temperature was determined by fitting the curve. (D) The photo images of e-skin being tested in a constant temperature and humidity box under specific conditions (25.2°C, 70% RH). (E) The instantaneous electrical response of the humidity-sensing layer. (F) The corresponding humidity was determined by fitting the curve.

Figure S19. (A) SEM images of the B-C-N PVA/PVDF electrospun nanofibers under different solution flow rates. (B) SEM images of the B-C-N PVA/PVDF electrospun nanofibers under different solution flow rates. (C) SEM images of the B-C-N PVA/PVDF electrospun nanofibers under different applied voltages. It can be seen from the figure that in order to obtain uniform nanofiber membrane, the optimal choice is: 15 wt.%, 25 kV and 1.5 mL $h^{-1}$.

Figure S20. (A) SEM images of the CA electrospun nanofibers under different solution flow rates. (B) SEM images of the CA electrospun nanofibers under different solution flow rates. (C) SEM images of the CA electrospun nanofibers under different applied voltages. It can be seen from the figure that in order to obtain uniform nanofiber membrane, the optimal choice is: 10 wt.%, 30 kV and 2 mL $h^{-1}$. After comparing Figure 1E and 1F, it is concluded that the process conditions are also suitable for CA-P-M.

Figure S21. (A) SEM images of the CA-M electrospun nanofibers under different solution flow rates. (B) SEM images of the CA-M electrospun nanofibers under different solution flow rates. (C) SEM images of the CA-M electrospun nanofibers under different applied voltages. It can be seen from the figure that in order to obtain uniform nanofiber membrane, the optimal choice is: 10 wt.%, 18 kV and 1 mL $h^{-1}$.

Table S1. Summary of the sensitivity of the pressure sensors.
Table S2. Summary of the sensitivity of the temperature sensors.
Table S3. Summary of the sensitivity of the humidity sensors.
Legends for movies S1 to S2.

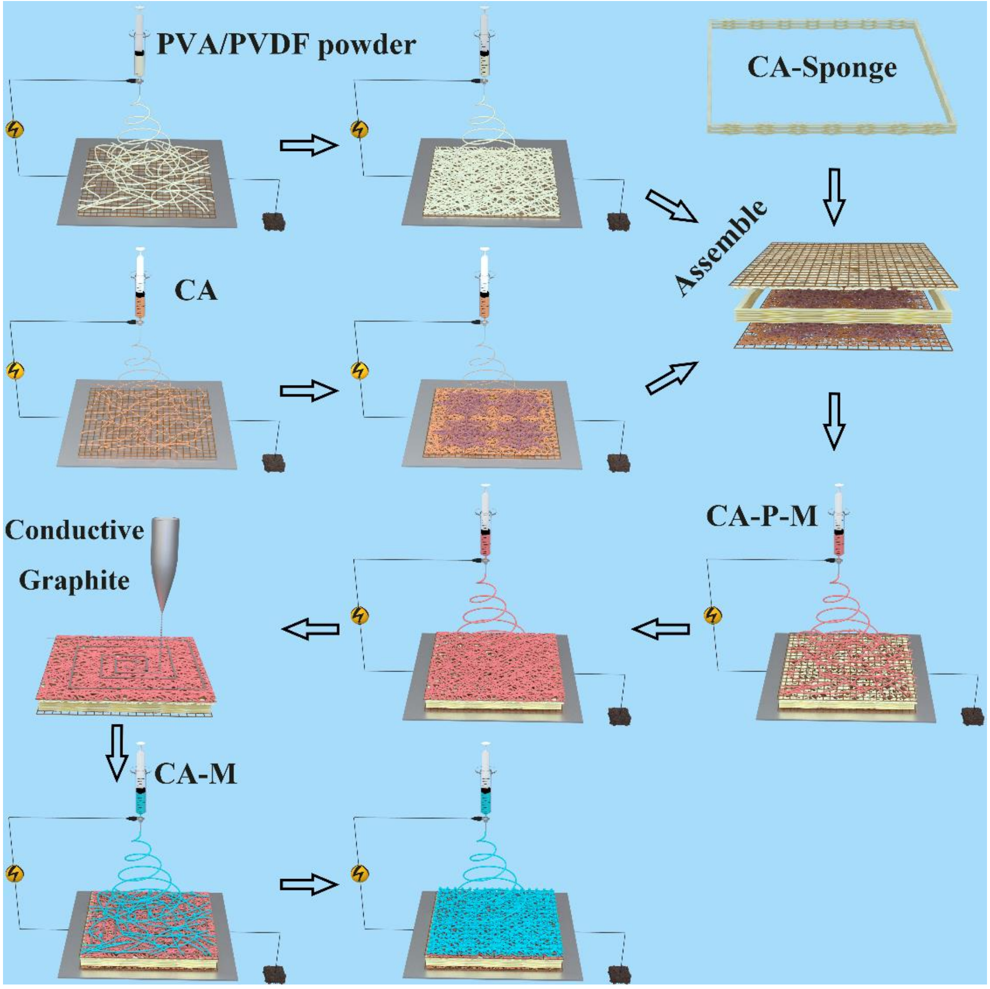


**Figure S1.** Preparation process of intelligent integrated e-skin.

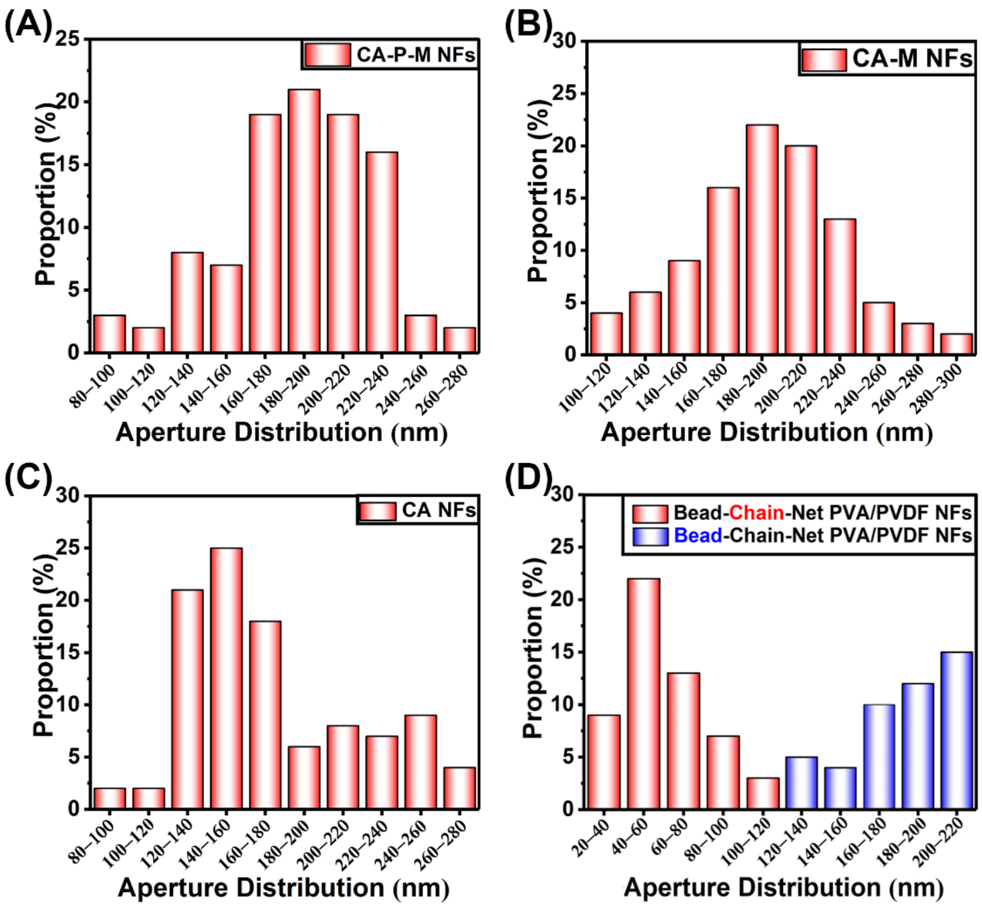


**Figure S2.** (A) Diameter distribution of the CA-M NFs. (B) Diameter distribution of the CA-P-M NFs. (C) Diameter distribution of the CA NFs. (D) Diameter distribution of the Bead-Chain-Net PVA/PVDF NFs.

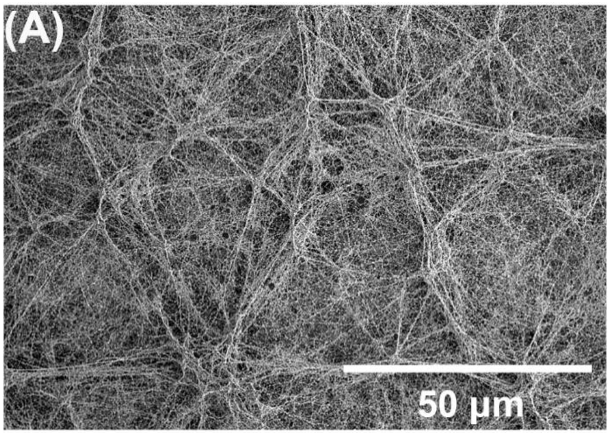


**Figure S3.** (A) SEM image of Bead-Chain-Net PVA/PVDF nanofibers.

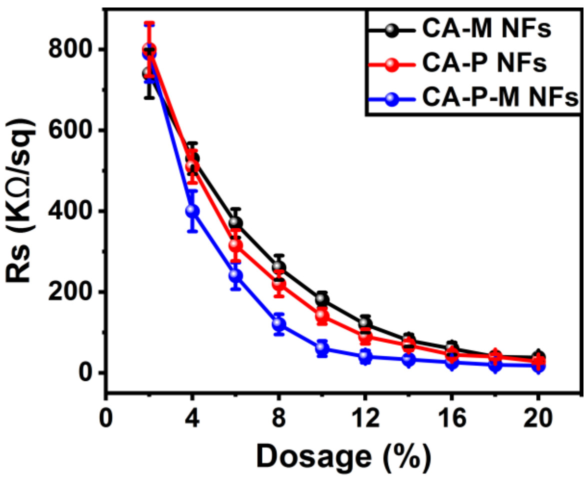


**Figure S4.** Sheet resistance of CA-M NFs, CA-P NFs and CA-P-M NFs after doping with different proportions of MWNTs, PEDOT: PSS and PEDOT-MWNTs.

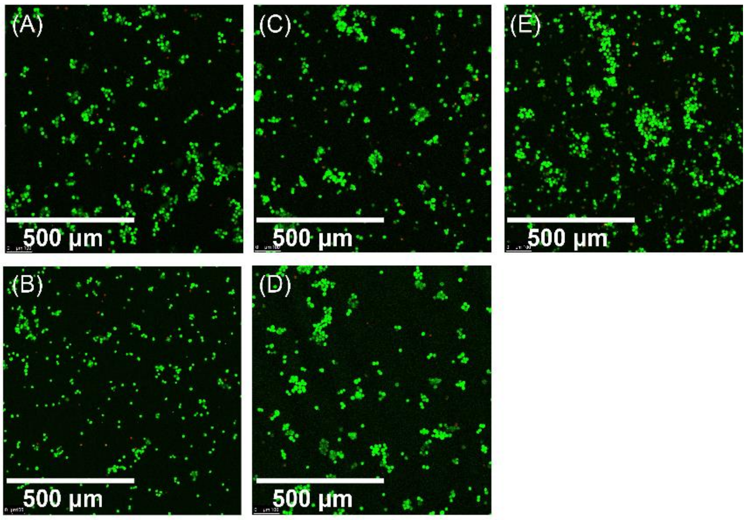


**Figure S5.** Photomicrograph of the cell density of the control (A, C and E) and e-skin (B, D) in MTT test on the first day (A, B), the third day (C, D), and the fifth day (E).

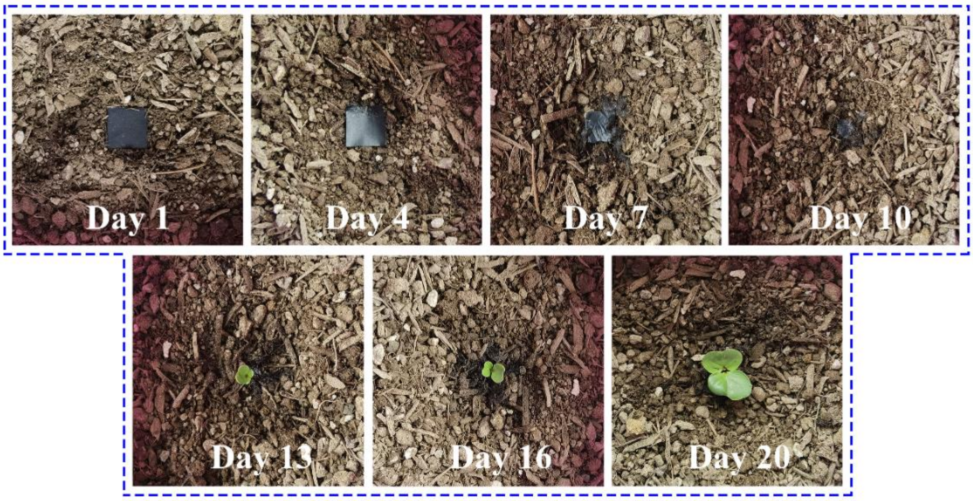

**Figure S6**. The natural degradation of e-skin occurred in the range of temperature and humidity (17 ± 2 °C, 55 ± 5%).

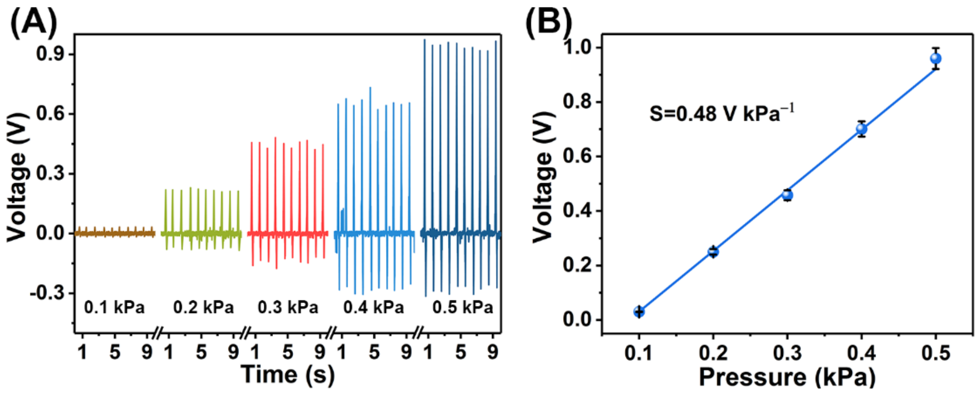


**Figure S7.** The signals of pressure-sensing layer under light pressure. (B)The sensitivity of e-skin pixels (1 × 1 $cm^2$) under light pressure.

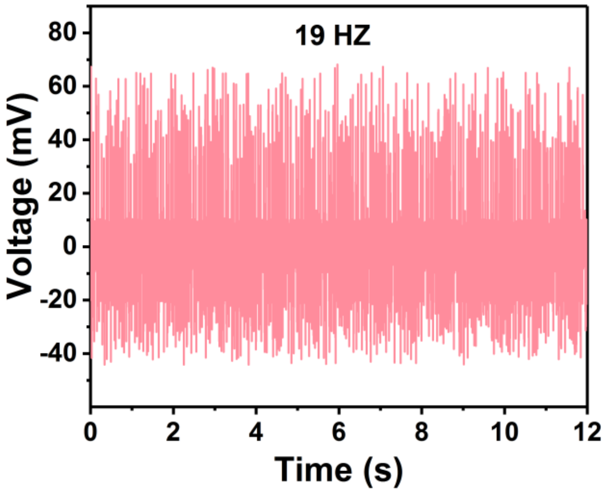


**Figure S8**. The signals of e-skin at 19 HZ.

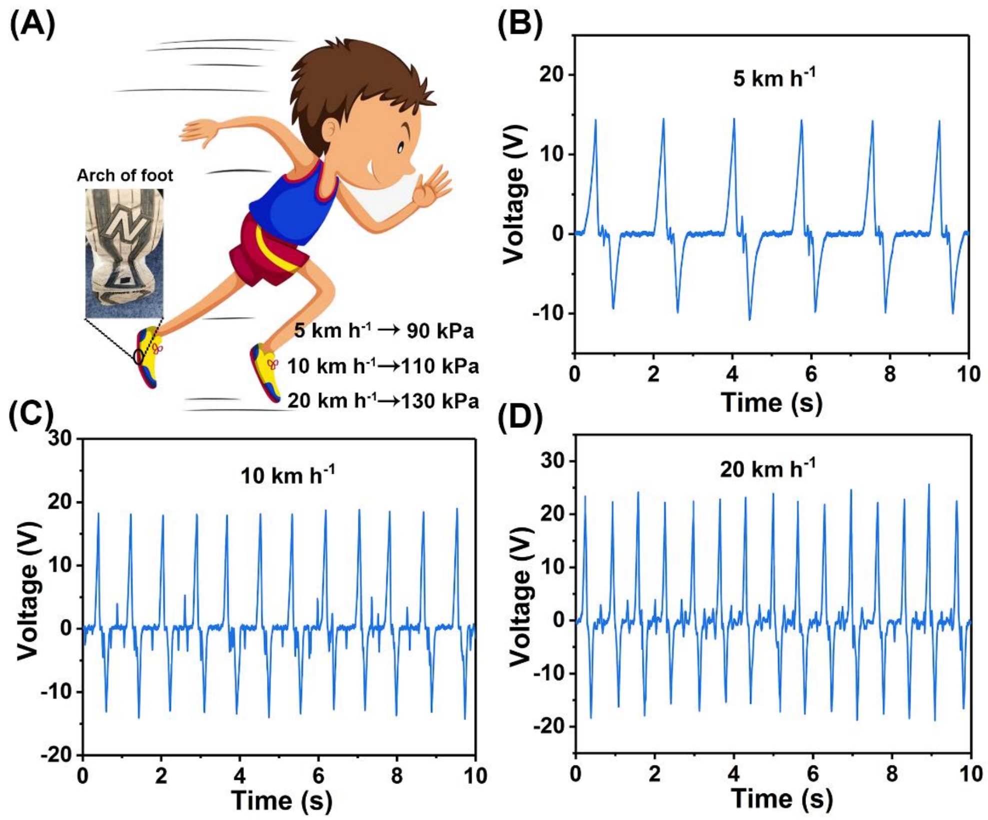


**Figure S9**. The signals of e-skin under high pressure: (A) The diagram of e-skin attached to the arch of foot to test the performance under different pressures caused by the tester running at different speeds (5 km $h^{-1}$,10 km $h^{-1}$, and 20 km $h^{-1}$ correspond approximately to 90 kPa, 110 kPa, and 130 kPa, respectively). (B) The signals of e-skin when the tester run at speed of 5 km $h^{-1}$. (C) The signals of e-skin when the tester run at speed of 10 km $h^{-1}$. (D) The signals of e-skin when the tester run at speed of 20 km $h^{-1}$.

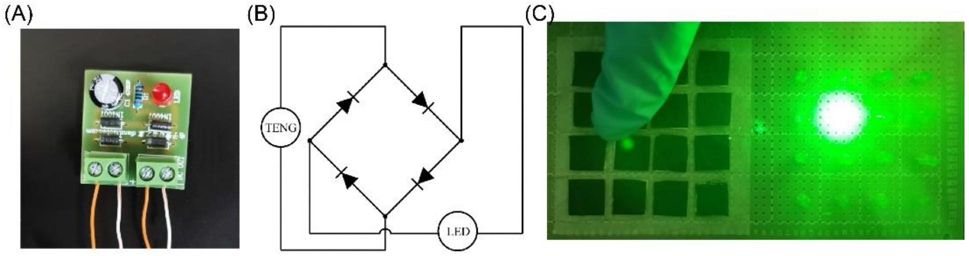


**Figure S10.** (A) Sample photos of commercial bridge rectifiers. (B) Circuit schematic diagram of commercial bridge rectifier. (C) Photo of the current produced by the e-skin driving the LED after being processed by a commercial bridge rectifier.

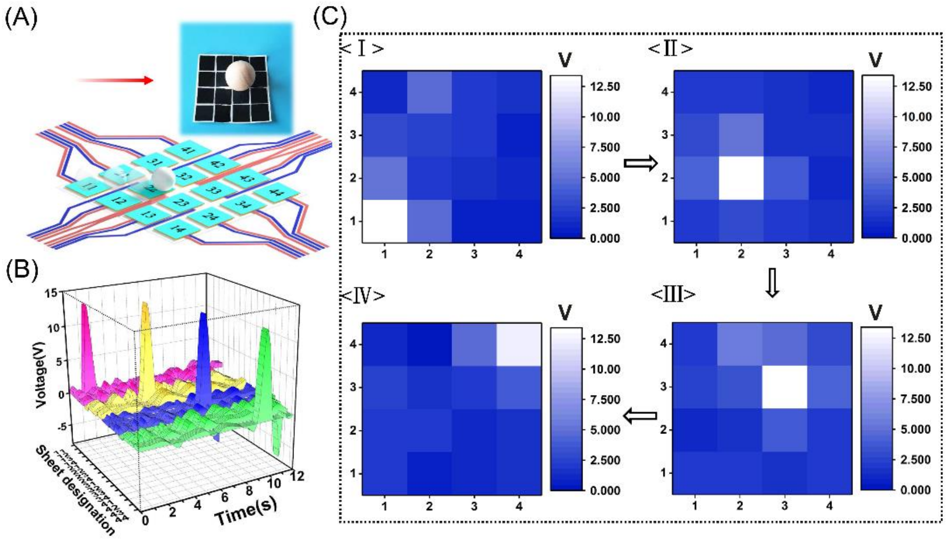

**Figure S11.** (A) An explanatory drawing of the movement of a 30 g ball in e-skin from "11" to "44". Optical photo inside. (B and C) Corresponding signals during the movement of the ball.

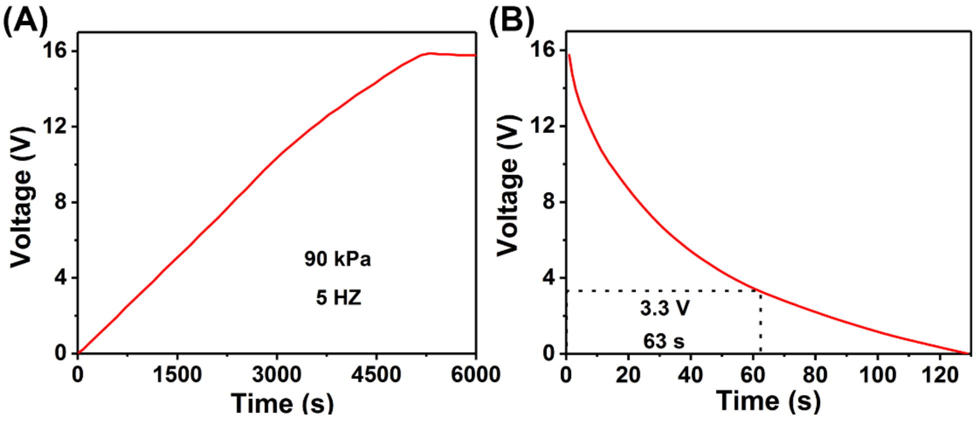


**Figure S12.** A single e-skin pixel charges a commercial capacitor (16V, 1000 μF) under certain conditions (90 kPa, 5 HZ). (B) The capacitor acted as the power source for the humidity and temperature detection of e-skin.

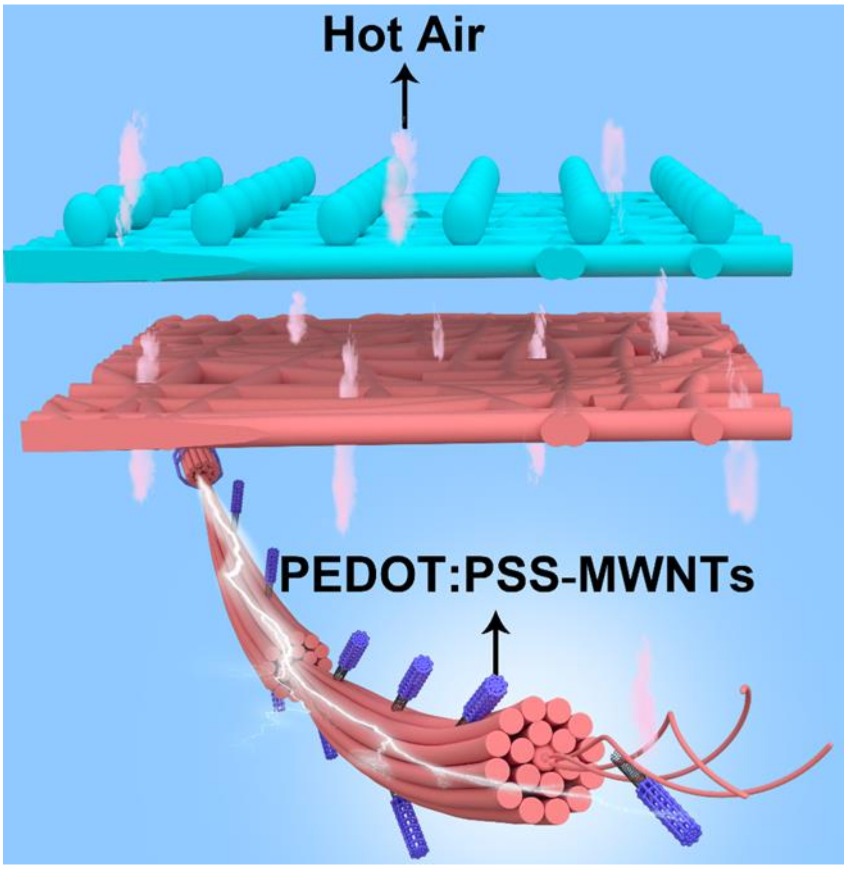


**Figure S13.** The mechanism of temperature-sensing layer to temperature.

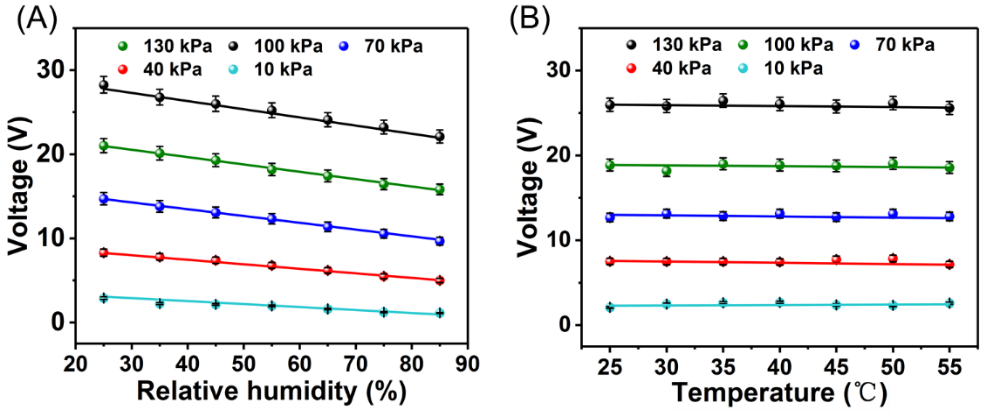


**Figure S14.** (A) The influence of the pressure-sensing layer under different relative humidity at 25°C. (B) The influence of the pressure-sensing layer under different temperature at 45% RH.

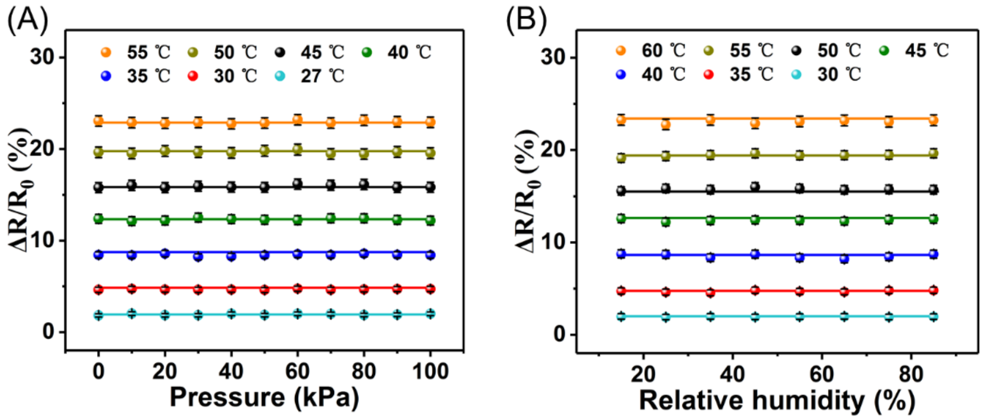


**Figure S15.** (A) The influence of the temperature-sensing layer under different pressures at 45% RH. (B) The influence of the temperature-sensing layer under different humidity at 130 kPa.

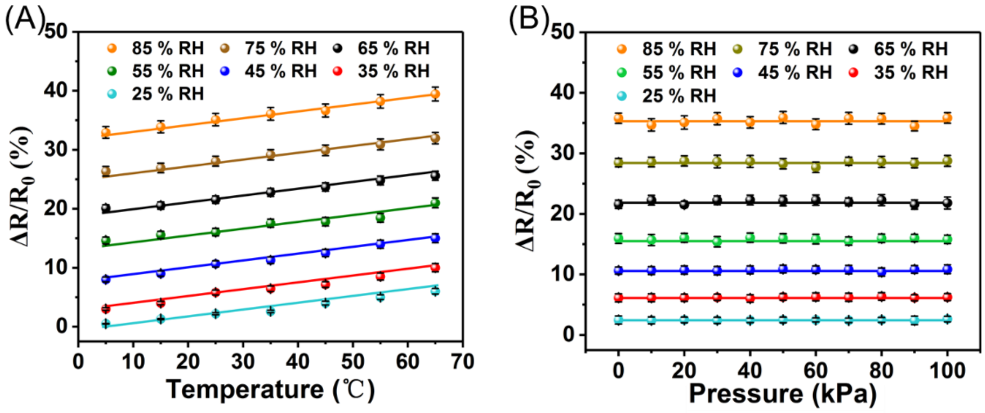


**Figure S16.** (A) The influence of the humidity-sensing layer under different temperature at 130 kPa. (B) The influence of the humidity-sensing layer under different pressure at 25°C.

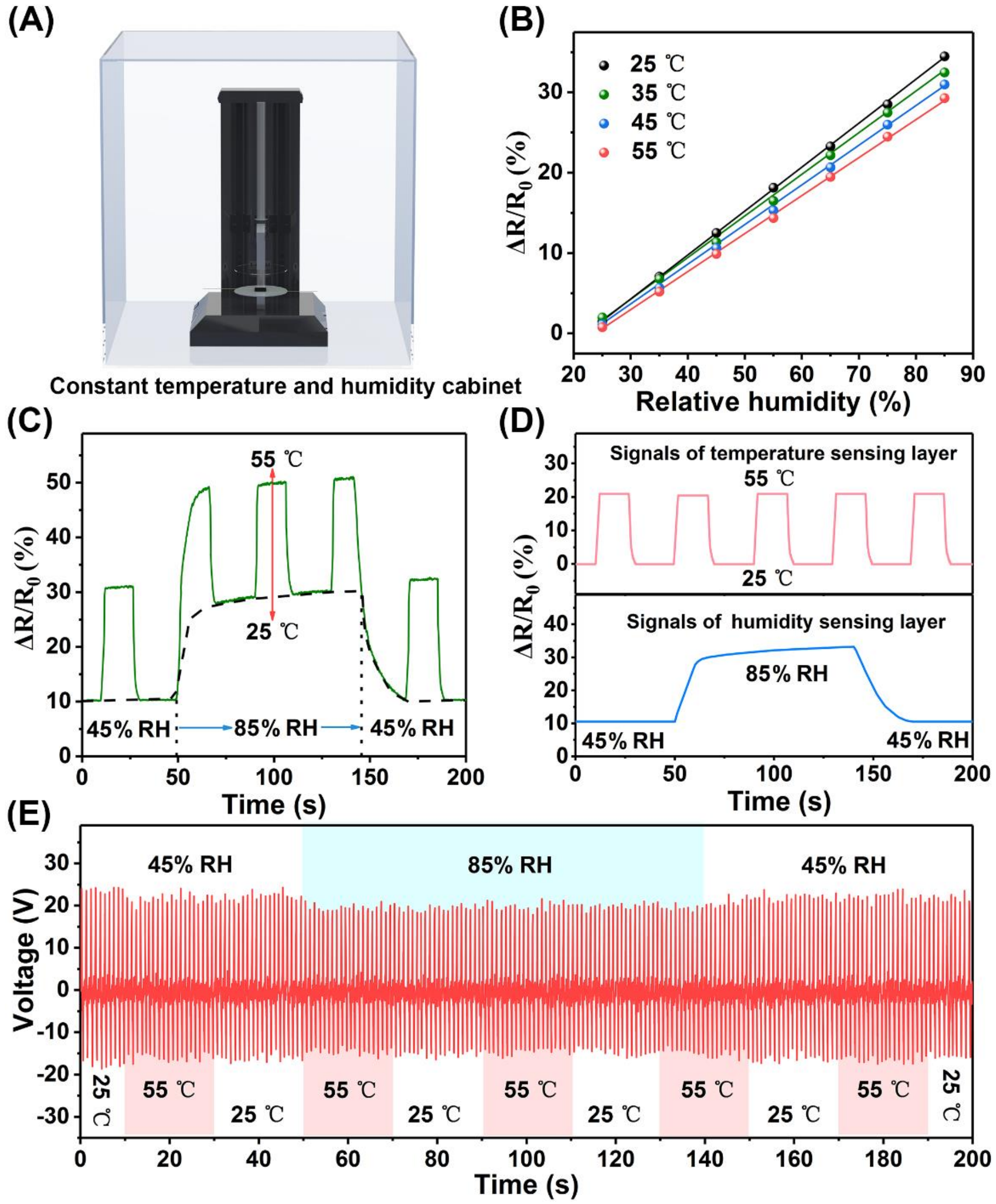


**Figure S17.** (A) Illustration of e-skin tested in a constant temperature and humidity cabinet. (B) The signals fitting and compensation of temperature sensor layer at different humidity. (C) (E) The signals of e-skin in complex conditions (25/55°C, 45/85% RH, and 0/130 kPa). (D) Temperature and humidity signals after separation and compensation.

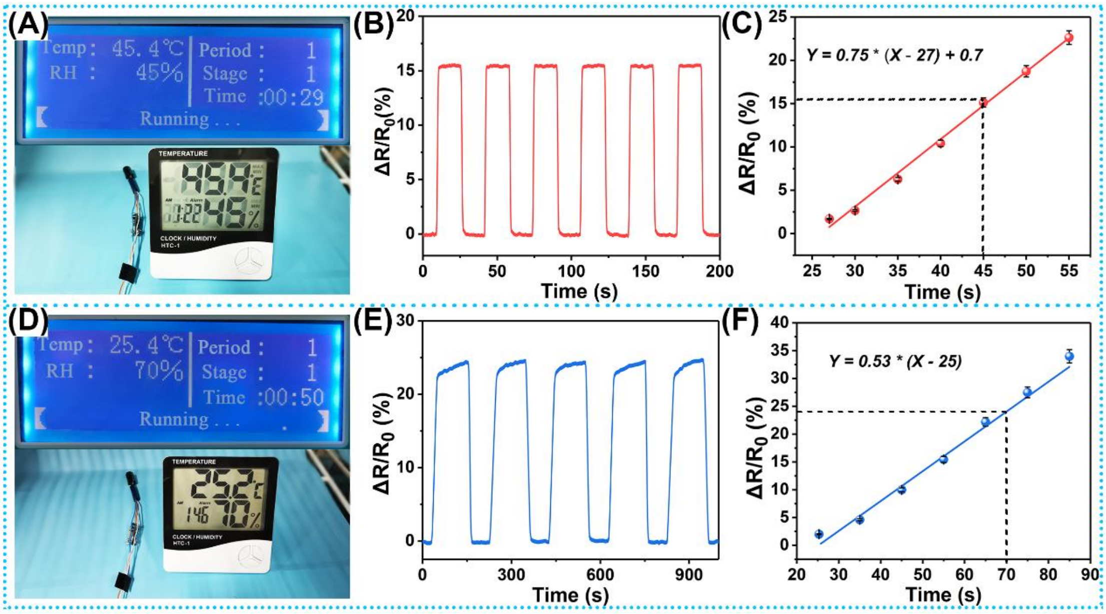


**Figure S18.** (A) The photo images of e-skin being tested in a constant temperature and humidity box under specific conditions (45.4°C, 45% RH). (B) The instantaneous electrical response of the temperature-sensing layer. (C) The corresponding temperature was determined by fitting the curve. (D) The photo images of e-skin being tested in a constant temperature and humidity box under specific conditions (25.2°C, 70% RH). (E) The instantaneous electrical response of the humidity-sensing layer. (F) The corresponding humidity was determined by fitting the curve.

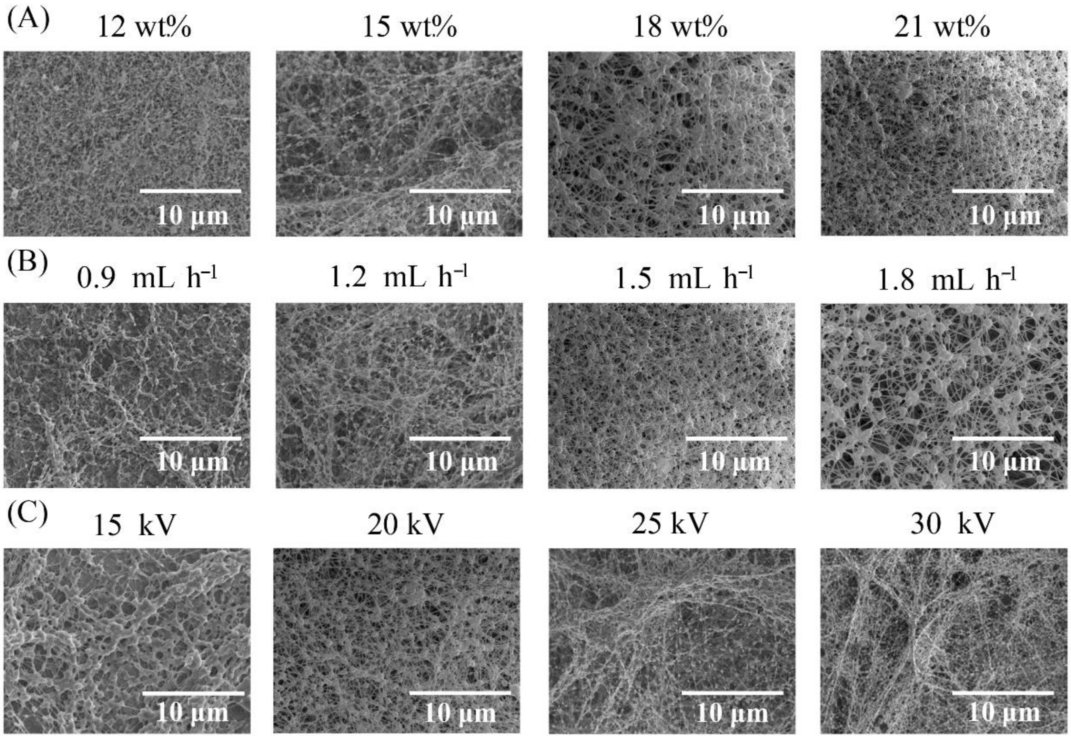


**Figure S19.** (A) SEM images of the B-C-N PVA/PVDF electrospun nanofibers under different solution flow rates. (B) SEM images of the B-C-N PVA/PVDF electrospun nanofibers under different solution flow rates. (C) SEM images of the B-C-N PVA/PVDF electrospun nanofibers

under different applied voltages. It can be seen from the figure that in order to obtain uniform nanofiber membrane, the optimal choice is: 15 wt.%, 25 kV and 1.5 mL $h^{-1}$.

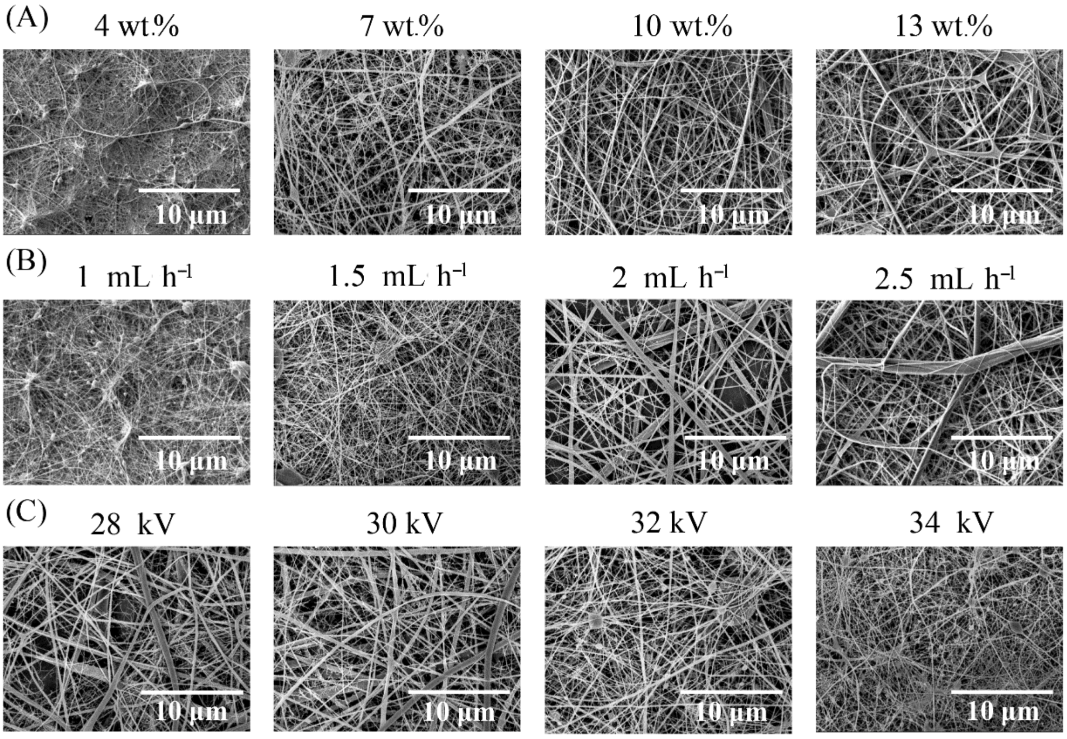


**Figure S20.** (A) SEM images of the CA electrospun nanofibers under different solution flow rates. (B) SEM images of the CA electrospun nanofibers under different solution flow rates. (C) SEM images of the CA electrospun nanofibers under different applied voltages. It can be seen from the figure that in order to obtain uniform nanofiber membrane, the optimal choice is: 10 wt.%, 30 kV and 2 mL $h^{-1}$. After comparing Figure 1E and 1F, it is concluded that the process conditions are also suitable for CA-P-M.

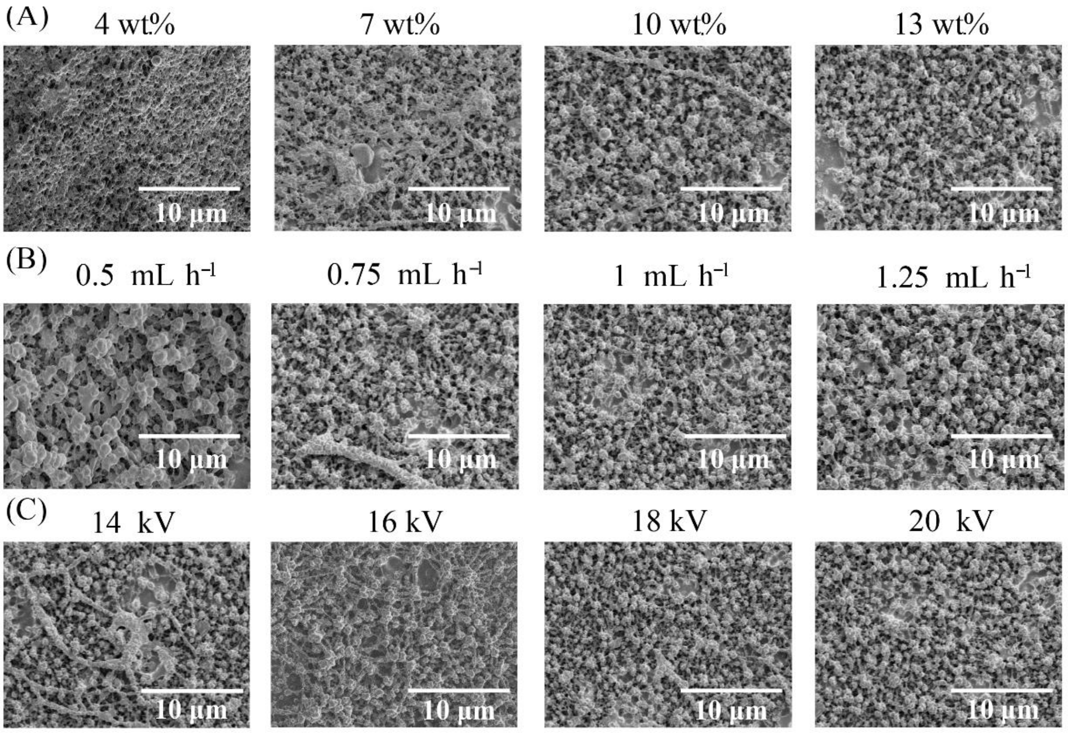

**Figure S21.** (A) SEM images of the CA-M electrospun nanofibers under different solution flow rates. (B) SEM images of the CA-M electrospun nanofibers under different solution flow rates. (C) SEM images of the CA-M electrospun nanofibers under different applied voltages. It can be seen from the figure that in order to obtain uniform nanofiber membrane, the optimal choice is: 10 wt.%, 18 kV and 1 mL $h^{-1}$.

**Table S1.** Summary of the sensitivity of the pressure sensors.

| Materials | Sensitivity (V $kPa^{-1}$) | Detection range (kPa) | Reference |
|---|---|---|---|
| PVDF/Ag NWs | 0.20 | 0–30 | [1] |
| PDMS/Ag | 0.06 | 0–90 | [2] |
| PZT | 0.018 | 0–30 | [3] |
| EVA/FEP | 0.25 | 0–40 | [4] |
| THV/COC | 0.030 | 0–150 | [5] |
| PLGA/PVA | 0.011 | 0–45 | [6] |
| CA/PVDF-PVA | 0.25 | 0–130 | This work |

**Table S2.** Summary of the sensitivity of the temperature sensors.

| Materials | TCR (°$C^{-1}$) | Sensing ranges (°C) | Reference |
|---|---|---|---|
| Pt | 0.00024 | 0–70 | [7] |
| PPy/Ag NWs | 0.00086 | 17–50 | [8] |
| Ag NFs/Ag NWs | 0.0003 | 30–45 | [9] |
| CNTs/InGaZnO | 0.0068 | 22.4–40 | [10] |
| PEDOT/Graphene | 0.006 | 35–45 | [11] |
| PEDOT/PDMS | 0.0044 | 30–55 | [12] |
| CA-P-M NFs | 0.0075 | 25–55 | This work |

**Table S3.** Summary of the sensitivity of the humidity sensors.

| Materials | Res and rec time (s) | Range (% RH) | Reference |
|---|---|---|---|
| Paper/PET conductive tapes | 472, 19 | 41.1–91.5 | [13] |
| Au/ZnO | 55, 100 | 11–95 | [14] |
| PANI | 760, 170 | 11–95 | [15] |
| Graphene | 200, 150 | 20–90 | [16] |
| PI | 18, 31 | 5–85 | [17] |
| CNTs/paper | 333, 523 | 11–95 | [18] |
| MWNTs/CA | 16, 25 | 25–85 | This work |

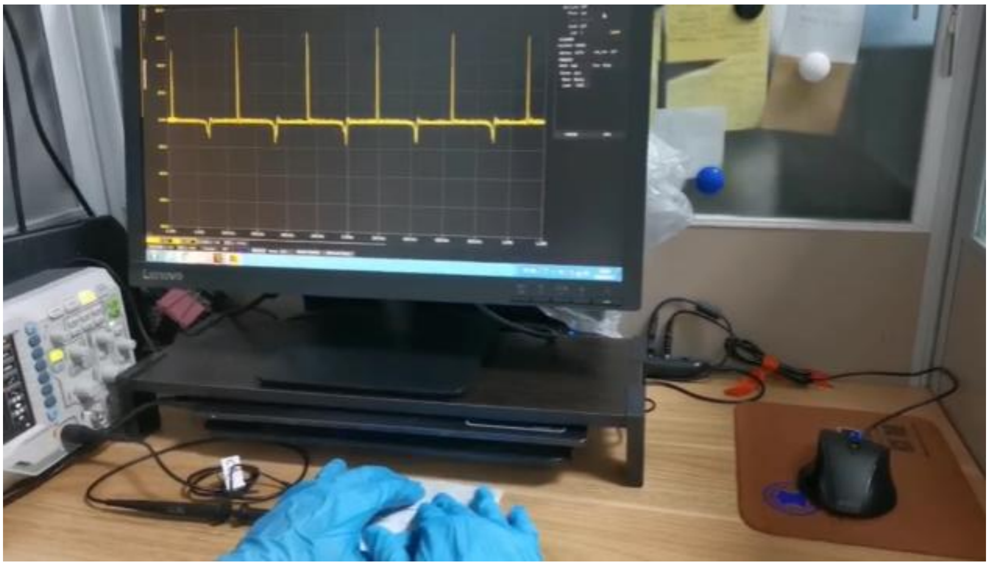

**Video S1.** Instantaneous signals of a pixel (1 × 1 $cm^2$) of e-skin when fingers were pressed.

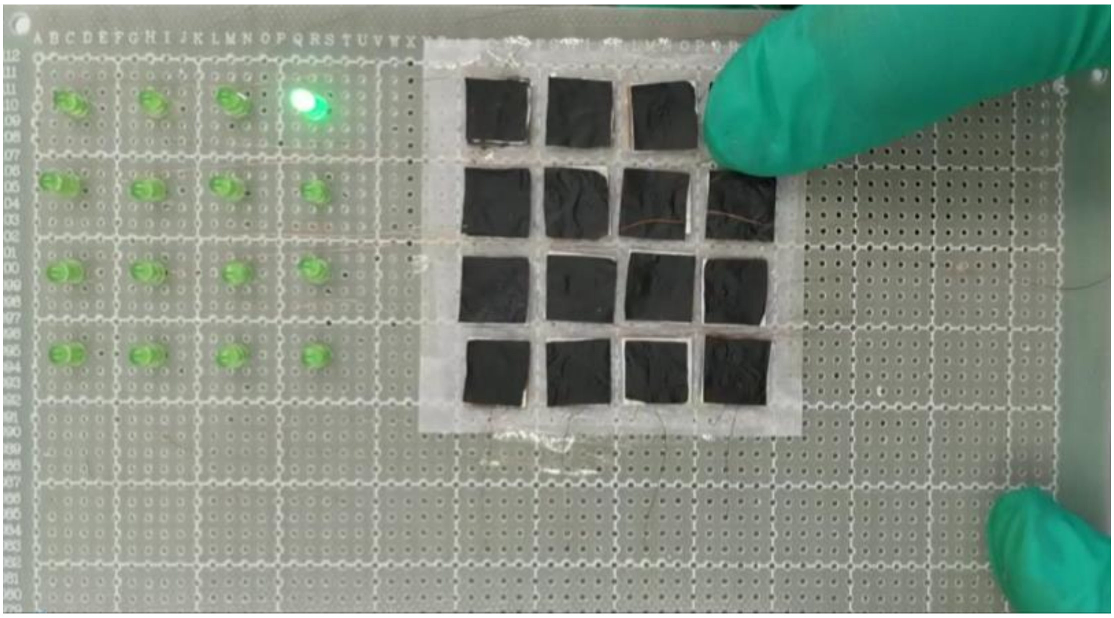

**Video S2.** Driving the LED by pressing the pixel of e-skin with finger.

## REFERENCES


[1] M. Lou, I. Abdalla, M. Zhu, J. Yu, Z. Li, and B. Ding, *ACS Appl. Mater. Interfaces*. **2019**, 12, 1597-1605.

[2] X. Wang, H. Zhang, L. Dong, X. Han, W. Du, J. Zhai, C. Pan, and Z. L. Wang, *Adv. Mater.* **2016**, 28, 2896-2903.

[3] D. Y. Park, D. J. Joe, D. H. Kim, H. Park, J. H. Han, C. K. Jeong, H. Park, J. G. Park, B. Joung, and K. J. Lee, *Adv. Mater.* **2017**, 29, 1702308.

[4] S. Chen, N. Wu, L. Ma, S. Lin, F. Yuan, Z. Xu, W. Li, B. Wang, and J. Zhou, *ACS Appl. Mater. Interfaces*. **2018**, 10, 3660-3667.

[5] W. Li, J. Duan, J. Zhong, N. Wu, S. Lin, Z. Xu, S. Chen, Y. Pan, L. Huang, and B. Hu, *ACS Appl. Mater. Interfaces*. **2018**, 10, 29675-29683.

[6] X. Peng, K. Dong, C. Ye, Y. Jiang, S. Zhai, R. Cheng, D. Liu, X. Gao, J. Wang, and Z. L. Wang, *Sci. Adv.* **2020**, 6, eaba9624.
[7] Q. Hua, J. Sun, H. Liu, R. Bao, R. Yu, J. Zhai, C. Pan, and Z. L. Wang, *Nat. Commun.* **2018**, 9, 1-11.
[8] W. He, G. Li, S. Zhang, Y. Wei, J. Wang, Q. Li, and X. Zhang, *Acs Nano*. **2015**, 9, 4244-4251.
[9] B. W. An, S. Heo, S. Ji, F. Bien, and J.-U. Park, *Nat. Commun.* **2018**, 9, 1-10.
[10] W. Honda, S. Harada, S. Ishida, T. Arie, S. Akita, and K. Takei, *Adv. Mater.* **2015**, 27, 4674-4680.
[11] T. Vuorinen, J. Niittynen, T. Kankkunen, T. M. Kraft, and M. Mäntysalo, *Sci. Rep.* **2016**, 6, 1-8.
[12] Y. Yu, S. Peng, P. Blanloeuil, S. Wu, and C. H. Wang, *ACS Appl. Mater. Interfaces*. **2020**, 12, 36578-36588.
[13] Z. Duan, Y. Jiang, M. Yan, S. Wang, Z. Yuan, Q. Zhao, P. Sun, G. Xie, X. Du, and H. Tai, *ACS Appl. Mater. Interfaces*. **2019**, 11, 21840-21849.
[14] S. Yu, H. Zhang, C. Chen, and C. Lin, *Sensors and Actuators B: Chemical*. **2019**, 287, 526-534.
[15] H. Ryu, S. J. Cho, B. Kim, and G. Lim, *RSC advances*. **2014**, 4, 39767-39770.
[16] D. H. Ho, Q. Sun, S. Y. Kim, J. T. Han, D. H. Kim, and J. H. Cho, *Adv. Mater.* **2016**, 28, 2601-2608.
[17] J. Boudaden, M. Steinmaßl, H.-E. Endres, A. Drost, I. Eisele, C. Kutter, and P. Müller-Buschbaum, *Sensors*. **2018**, 18, 1516.
[18] P. Zhu, Y. Kuang, Y. Wei, F. Li, H. Ou, F. Jiang, and G. Chen, *Chem. Eng. J.* **2020**, 404, 127105.
